# A Comprehensive Review of Plant Volatile Terpenoids, Elucidating Interactions with Surroundings, Systematic Synthesis, Regulation, and Targeted Engineering Production

**DOI:** 10.3390/biology14050466

**Published:** 2025-04-25

**Authors:** Wei Jin, Zhongzhou Yang, Kedong Xu, Qiuping Liu, Qi Luo, Lili Li, Xiaohong Xiang

**Affiliations:** 1 College of Life Science and Agriculture, Zhoukou Normal University, Zhoukou 466001, China; jinw877@126.com (W.J.); liuqp06@163.com (Q.L.); lilili@zknu.edu.cn (L.L.); 2 Field Observation and Research Station of Green Agriculture in Dancheng County, Zhoukou 466001, China; 3 Key Laboratory of Crop Molecular Breeding and Bioreactor, Zhoukou Normal University, Zhoukou 466001, China; yangzz121@126.com (Z.Y.); xukd1107@126.com (K.X.); roach07@163.com (Q.L.); 4 Henan International Joint Laboratory of Translational Biology, Zhoukou Normal University, Zhoukou 466001, China; 5 School of Pharmacy, Chongqing Medical and Pharmaceutical College, Chongqing 401331, China

**Keywords:** volatile terpenoids, terpenoid synthase genes, regulatory factors, target engineering

## Abstract

Plants require a flexible avoidance mechanism as they need to cope with external stimuli and challenges through complex specialized metabolites, among which volatile terpenoids make outstanding contributions, acting as key media signal substances in the cooperation between plants and surrounding organisms. In recent decades, the research on the identification and functional characterization of terpenoid synthase and factors regulating metabolic shunts has gained significant attention, leading to substantial progress and notable achievements. However, with the popularization of terpenoids in insect and disease prevention, medical care, cosmetics, and other fields, coupled with increasing resistance to artificially produced chemical products, the demand for natural terpenoids has outpaced supply, prompting the emergence and popularity of targeted engineering for the mass production of terpenoids using microorganisms and plants as platforms. In this paper, we provide a detailed overview of the key knowledge and research progress of volatile terpenoids with regard to multiple functions, complex synthetic pathways, key terpenoid synthase genes, related regulatory factors, and target engineering.

## Simple Summary

Volatile terpenoids play a vital role as key signaling substances in the construction of flexible defense mechanisms in plants and their collaboration with surrounding organisms. With their wide biological properties, they are widely used in pest control, healthcare, food, cosmetics, etc. As the demand for natural terpenoids surges, targeted production engineering using microorganisms and plants as platforms has emerged. This paper comprehensively reviews the research progress of volatile terpenoids with regard to functions, synthetic pathways, key genes, regulatory factors, and targeted engineering, providing an important theoretical basis for terpenoid engineering production and the molecular breeding of target terpenoid plants.

## 1. Introduction

Plants have undergone a prolonged evolutionary transition from aquatic to terrestrial. Angiosperms originated approximately 100 million years ago and rapidly became the most widespread and diverse plants on earth. The floral organs of angiosperms represent one of the most critical characteristics distinguishing them from other plants. The emergence of floral organs marked a pivotal adaptation to the terrestrial environment, ensuring successful reproduction in angiosperms and enhancing the adaptability and subsequent proliferation of early terrestrial plants [1]. Angiosperm flowers synthesize and emit a wide variety of volatile organic compounds (VOCs), including terpenoids, esters, alcohols, alkanes, and olefin, which play crucial roles in plant–plant and plant–insect interactions, such as attracting insect pollination and inducing plant self-defense [2,3,4]. Terpenoids are among the most significant components of floral volatiles. To date, over 556 terpenoids have been identified in floral volatiles [5]. In ornamental plants, volatile terpenoids directly influence their fragrance and aesthetic value. In addition, terpenoids, as important secondary metabolites of plants, play key roles in regulating plant growth and development, responding to environmental changes, and combating pathogenic microorganisms, pests, and diseases [6,7,8]. In addition, terpenoids possess a wide range of biological activities that are crucial for human health, such as anti-inflammatory, bactericidal, antioxidant, and antidepressant effects, and are widely used in the medical field [9]. For example, terpenoids have been proven to have the potential to be used as chemotherapeutic agents for treating tumors [10]. It was found that Myrcene and α-Pinene can play a role in cancer treatment by inducing apoptosis and reducing cell growth, respectively [11,12]. Limonene, mainly produced in citrus fruits, has shown significant cytotoxic effects on various cancer cells [13]. Terpenoids also have a neuroprotective effect by reducing inflammation. Montenegro et al. found that terpenoids in olive leaf extracts have neuroprotective potential [14]. Xu et al. pointed out that 33 monoterpenoids, including linalool, menthol, α-limonene, and α-terpineol, have great potential for applications in the prevention or treatment of neurological disorders [15]. Many terpenoids, such as carvone, linalool, and menthol, have been proven to have antibacterial effects [9]. Myrcene has been confirmed to have anti-inflammatory, analgesic, and antibacterial activities, menthol acts as a cooling agent and can relieve pain more effectively [16], and linalool has a pleasant smell and can be used to treat anxiety [17,18]. It is worth noting that terpenoids can act as local irritants and may cause toxic reactions such as gastrointestinal symptoms, altered mental status, seizures, and even comas due to excessive intake. In addition, terpenoids such as limonene, linalool, and α-pinene can enhance the sensory quality of products and extend the shelf life of food, so they are widely used in the fields of cosmetics and food additives, which are closely related to human health [9]. Although the impacts of terpenoids on human health and their specific mechanisms of action are still far from being fully understood, there is no doubt about the significant value of terpenoids in maintaining human health. Therefore, research on terpenoid synthesis and metabolism has remained a key focus for scientists.

Terpenoids are derived from isophthalic acid, with isoprene units (C5 units) as the basic structural units in the molecular skeleton, and are extensively synthesized in almost all plant organs, including the leaves, roots, stems, flowers, fruits, and seeds, with particularly high concentrations in the flowers. Approximately 20,000 terpenoids have been identified and structurally characterized by scientists. Terpenoids are further subdivided into monoterpenes (C10) (e.g., linalool, limonene, and terpinene), sesquiterpenes (C15) (e.g., caryophyllene, artemisinin, and gemene), diterpenes (C20) (e.g., ricinolene and gibberellin,), triterpenes (C30) (e.g., ginsenosides, soybean saponins, and sterols), and irregular terpenes (>C30). This classification is based on the number of repeating units of isoprene, a five-carbon molecule that serves as the structural hallmark of all terpenes [19]. Generally, certain monoterpenes, sesquiterpenes, and diterpenes are crucial for plant–organism interactions, while triterpenoids and chlorophyll contribute to photosynthesis and ubiquinones are involved in plant respiration. Among these terpenoids, monoterpenoids and sesquiterpenoids exhibit volatile properties that affect the fragrance profiles of flowers and fruits of many plants. In nature, monoterpenes are significantly more abundant than sesquiterpenes. For example, in common plant families such as Apiaceae, Labiaceae, and Asteraceae, the volatile monoterpene linalool is released in substantial quantities [20,21,22]. Monoterpenoids typically constitute over 80% of the composition of most plant essential oils [23,24,25]. Reports indicate that monoterpenes and sesquiterpenes comprise 53% and 28% of total volatiles in the flowers of most plants, whereas diterpenes and triterpenes contribute less than 1% [26,27,28].

## 2. Interactions with Surroundings

**Figure 1:**
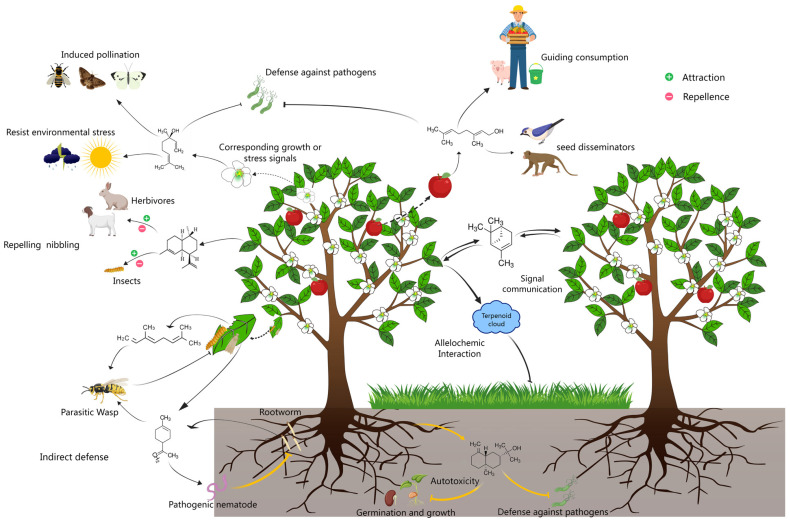
Schematic showing plant volatile terpenoid-mediated interactions between plants and surrounding organisms. In addition to granting plants thermotolerance and photoprotection in response to stress signals, volatile terpenes confer multiple functions to plants through interactions with their surrounding organisms. In most cases, these comprise direct interactions, such as attracting pollinators and seed disseminators, repelling/attracting herbivores and insects, and resisting pathogen invasion, autotoxicity, and allelochemic activity from competitive nearby plants, and indirect effects, including attracting the natural enemies of aggressors, triggering defense alerts in plants without incidents as a signal communication medium, and so on.

Terpenoids exhibit a rich diversity in terms of species, along with varied structures and functions. Despite the limited understanding of the spatiotemporal dynamics of their perceptual signaling, it is widely recognized that terpenoids play critical roles in mediating plant–plant and plant–environment communication (Figure 1). Recent research has begun to address this knowledge gap, demonstrating that volatiles released by injured plants are absorbed through the stomata of healthy leaves, triggering a rapid increase in cytosolic calcium ([Ca^2+^]_cyt_) levels to initiate a defense response [29].

### 2.1. Security Pollination

With the diversification and proliferation of plants, particularly angiosperms, numerous cross-pollinated species emerged and rapidly expanded through wind, insect, and bird vectors. Furthermore, competitive phenotypes, commonly referred to as pollination syndromes, which are characterized by specialized flower structures, vibrant colors, and rich aromas, have evolved to optimize this cross-pollination strategy, ensuring efficient pollen transfer. For example, the morphological structures of columbine flowers influence the types of pollinators they attract [30]. The color change of *Lotus corniculatus* occurs after pollination, which causes a negative frequency of bee visits and prevents superfluous pollination [31]. Many flowering plants attract insects by releasing substantial quantities of wind-dispersed volatiles, significantly enhancing pollination efficiency [32,33].

Recent research indicates that volatile terpenoids serve as unique chemical signals that facilitate communication between plants and pollinators. These compounds attract specific pollinators by altering their components or proportions, thereby aiding in plant pollination. It has been reported that approximately 90% of the Earth’s angiosperms rely on floral assistance to complete pollination [34]. The presence of straight-chain trienoic acid, cineole, or geraniol in most plant pollens has been reported as a powerful attractant for bees and a key factor in their orientation [35]. Linalool, a common monoterpene compound, is present in nearly all floral volatiles. It functions as an insect attractant and can be captured by insects at extremely low concentrations. Additionally, it serves to indicate the location of nectar glands in flowers to pollinators [36]. With the development of collection and detection techniques for flower volatiles, it has been found that moth-pollinated plants release high quantities of phenylpropanoids, terpenoids, and nitrogenous compounds to attract target pollinators, while bird-pollinated plants predominantly emit terpenoids and aliphatic derivatives in flower volatiles [31]. Moreover, the attraction of different terpenoids to insects may serve as an important driving force for reproductive isolation and speciation. For instance, *Petunia axillaris* emits significant quantities of volatile terpenoids, attracting nocturnal moths for pollination, whereas *Petunia inregrifolia*, another variety of *Petunia*, emits fewer volatile compounds and relies on diurnal bees for pollination [37]. Variations in the concentrations of myrcene, ocimene, and limonene in the volatile compounds of *Mimulus lewisii* and *M. cardinalis* led to a shift in the pollination mode from bees to birds [6].

### 2.2. Plant Defense

Research on the role of terpenoids in plant defense began gaining attention in 1990, when scientists from the United States and the Netherlands first reported that certain plants release substantial amounts of volatile terpenoids to attract the predators of insect pests after being nibbled on [38]. To date, numerous studies have established that terpenoids function as signaling molecules, mediating plant defense responses to herbivorous insects and playing a critical role in defending against pathogenic microorganism invasion. Table 1 lists the literature citing volatile terpenoids involving plant defense.

The conclusion that plants emit significant quantities of terpenoids in response to insect gnawing is well-documented. For example, the gnawing of spotted spider mites induces the emission of (E)-β-Ocimene and transcript accumulation of (E)-β-Ocimene synthase in *Lotus japonicus* [39]. The expression of specific terpenoid synthase genes and their corresponding terpenoid products significantly increases when *Malacosoma disstria* feeds on poplar leaves [40]. Although the precise mechanism by which plants perceive terpenoid volatile signals remains poorly understood, they are generally regarded as airborne signals that trigger defense responses in neighboring plants [41,42]. Non-infested lima beans (*phaseolus lunatus*) are more effectively protected from the threat of spiders by triggering defense responses when exposed to volatiles emitted by their infested conspecifics [43].

Numerous reports indicated that terpenoids function as antifeedants and toxic agents, providing direct protection to plants against insect pests. Research has demonstrated that floral volatiles in warm tropical regions function as chemical signals to modulate ant behavior, preventing them from accessing functional flowers [44]. Monoterpenes, including limonene and myrcene, exhibit potent insecticidal properties [45]. Additionally, plants utilize terpenoids to attract the natural enemies of pests, thereby indirectly serving a defensive function. Rice plants infested by *Spodoptera frugiperda* caterpillars emit a volatile blend dominated by terpenoids, which strongly attract female parasitoid wasps [46]. Maize plants can release significant amounts of caryophyllene, a compound that provides indirect resistance against pests by attracting their natural enemies, through the upregulation of *terpene synthase* (*TPS*) genes after pest feeding, which attracts the natural enemies of these pets [47,48]. Initially, it was thought that physical trauma caused by insect gnaws was the inducement of plant defense functions at the outset. However, subsequent research revealed that insect oral secretions, rather than physical damage, play a pivotal role in inducing the terpenoid defense mechanism, compared with simple mechanical wounds. For example, the compound *N*-(17-hydroxylinolenoyl)-l-glutamine, isolated from beet armyworm caterpillar oral secretions, was identified as an elicitor, inducing corn seedlings dominated by typical terpenes including linalool, nerolol, and farnesene, making them highly attractive to parasitic wasps that prey on the caterpillars [49]. Cabbage leaves, when artificially damaged and subsequently infected with *P. brassicae* caterpillars’ regurgitant or commercial β-glucosidase, release a volatile blend that closely resembles the emissions of herbivore-damaged plants [50].

Terpenoids have been shown to benefit plants by defending against pathogenic microorganisms. Pathogenic bacteria are typically transmitted by insects and raindrops or spread with the wind, and persistently invade the plant’s reproductive and vegetative organs during the process of pollination [50]. Compared with plant tissues such as roots, stems, and leaves, flower organs, especially stigmas, lack a protective wood layer or cuticle layer and are rich in nutrients and moisture content, making them highly exposed to bacterial and fungal infection [51]. Bacteria populations’ survival on the stigma of a flower, as measured per gram, has been reported to be as high as 10^10^ colony-forming units [52]. How the flower organs of plants adapt to challenging environments has garnered significant attention from scientists. Numerous studies have confirmed that plant-derived volatile terpenoids serve as a decisive defense, controlling the growth of bacteria and fungi. For instance, caryophyllene-rich rhizome oil derived from *Zingiber nimmonii* has been shown to significantly inhibit the growth of *Bacillus subtilis* and *Pseudomonas aeruginosa* [53]. *Arabidopsis thaliana* lines exhibiting ectopic caryophyllene emissions demonstrated greater resistance to *Pseudomonas syringae* infection compared to wild-type plants [54]. Essential oils replicating the volatile composition of *Freesia* flowers showed antibacterial effects against a variety of fungi in vitro [55]. Thus, terpenoid volatiles emitted from floral tissues may function as a protective mechanism against microorganisms, potentially complementing or replacing their role in attracting pollinators.

It is worth mentioning that plants can enhance their survival competitiveness through allelopathy and autotoxicity, facilitating population expansion and contributing to natural selection in evolution. The dual effects of the low-concentration promotion and high-concentration inhibition of terpenoids are beneficial to the regulation of population density. When the plant population is small, stimulatory substances can promote plant reproduction and dispersal. However, as the population reaches a critical size, the inhibitory effects intensify due to the increased concentration of allelopathic substances, and the population size becomes restricted to the carrying capacity of the environment. *Artemisia californica* forms a “terpene cloud” around itself through the continuous volatilization of terpene substances from its leaves, which inhibits the seed germination and seedling growth of surrounding plants [56]. The terpenoids in plant essential oils, including limonene, pinene, camphor, and citronellol, have been shown to strongly inhibit seed germination and seedling growth [57]. The terpenoids released by Chinese fir (*Cunninghamia lanceolata*) exhibit pronounced autotoxicity [58].

**Table 1:** Volatile terpenoids are involved in plant defense responses.

Terpenoid	Defense Mechanism	References
(E)-β-Ocimene	In response to spotted spider mites gnawing	[39]
(-)-germacrene D	Increases significantly when *Malacosoma disstria* feeds on poplar leaves	[40]
Volatile terpenoids mixture	*Tetranychus urticae* nibbling induces defense TPS gene activation in neighboring lima bean leaves.	[43]
Volatile terpenoids mixture	Act as signals to prevent ants from approaching the flowers.	[44]
Limonene, myrcene	Possess insecticidal properties	[45]
Volatile terpenoids mixture	*Spodoptera frugiperda larvae* infested rice release a volatile terpenoids mixture to attract female parasitic wasps	[46]
Caryophyllene	corn plants upregulate terpene synthase (TPS), release caryophyllene to attract natural enemies of feeding insects	[47,48]
Linalool, Nerol, Farnesene	Attract parasitic wasps, the natural enemies of caterpillars	[49]
Volatile terpenoids mixture	*P. brassicae caterpillars* regurgitant induce terpenoid mixtures release in Cabbage leaves	[50]
aryophyllene	Significantly inhibit the growth of *Bacillus subtilis* and *Pseudomonas aeruginosa*	[53]
Caryophyllene	Caryophyllene endows *Arabidopsis thaliana* with stronger resistance to *Pseudomonas syringae*	[54]
Linalool	Show antibacterial effects against various fungi	[55]
Volatile terpenoids mixture	*Artemisia californica* inhibits the seed germination and seedling growth of surrounding plants by releasing a “terpene cloud”	[56]
Limonene, Pinene, Camphor, Citronellol	Autotoxicity /strongly inhibit seed germination and seedling growth	[57]
Volatile terpenoids mixture	The terpenoids released by Chinese fir exhibit obvious autotoxicity	[58]

## 3. Systematic Biosynthesis of Plant Terpenoids

### 3.1. Biosynthetic Pathway

**Figure 2:**
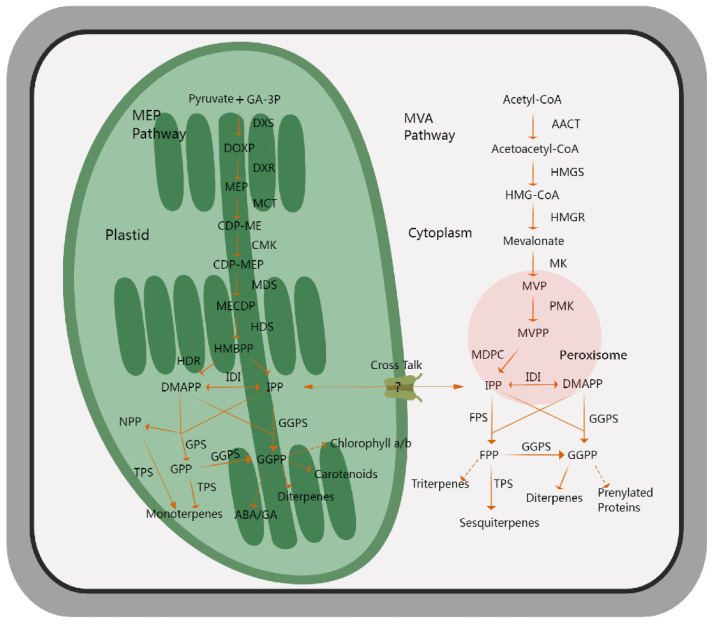
An outline showing the biosynthetic pathways of volatile terpenoids in plants. The MEP pathway, located in plastids, takes pyruvate and GA-3P as the initial substrates, undergoes a multi-step catalytic reaction, and mainly produces monoterpenes as the final product, while the MVA pathway, which is embedded in cytoplasm, mainly produces sesquiterpenes, as well as diterpenes and triterpenes, with acetyl-CoA as the initial substrates. The solid arrows indicate direct catalytic steps, and the dotted arrows represent multi-step catalytic processes. MEP, methylerythritol-4-phosphate; GA-3P, glyceraldehyde-3-phosphate; DXS, 1-deoxy-D-xylulose 5-phosphate synthase; DXR, 1-deoxy-D-xylulose 5-phosphate reductoisomerase; MCT, 2-C-methyl-D-erythritol 4-phosphate cytidylyltransferase; CMK, 4-(cytidine 59-diphospho)-2-C-methyl-D-erythritol kinase; MDS, 2-C-methyl-D-erythritol 2,4-cyclodiphosphate synthase; HDS, 4-hydroxy-3-methylbut-2-en-1-yl diphosphate synthase; DMAPP, dimethylallyl diphosphate; IPP, isopentenyl diphosphate; GPS, geranyl diphosphate synthase; NPP, nerol pyrophosphate; GPP, geranyl diphosphate; FPP, farnesyl diphosphate; ACCT, acetyl-CoA carboxylase; HMGS, 3-hydroxy-3-methylglutaryl-CoA synthase; HMGR, 3-hydroxy-3-methylglutaryl-CoA reductase; MK, mevalonate kinase; PMK, phosphomevalonate kinase; MVPP, pyrophosphate mevalonate; MVA, mevalonic acid; IDI, isopentenyl diphosphate isomerase; GGPS, geranylgeranyl pyrophosphate synthase; TPS, terpene synthase.

The synthetic pathway of terpenoids in plants has been well-elucidated after decades of research. Two terpenoid biosynthetic pathways, including the mevalerate (MVA) pathway and the methylerythritol-4-phosphate (MEP) pathway, have been categorized according to the differences in the subcellular interval and initial reaction substances [59,60,61]. The MVA pathway begins with acetyl-coenzyme A as the starting substance, undergoing enzymatic catalysis to produce the C5 precursor, isopentenyl diphosphate (IPP), or its isomeric form, dimethylallyl diphosphate (DMAPP). Subsequently, farnesyl diphosphate synthase (FPS) catalyzes the formation of farnesyl diphosphate (FPP), which serves as a direct precursor for the synthesis of terpene compounds, including sesquiterpenes and triterpenes [62]. Several studies have shown that β-methylglutarate monoacyl-coA reductase (HMGR) plays a rate-limiting function in this process [63,64]. The MEP pathway takes glycerol and pyruvate as the initial raw materials, is carried out in the plastid, and is catalyzed by various enzymes to form IPP and DMAPP. Further, geranyl diphosphate synthase (GPS) and geranylgeranyl diphosphate synthase (GGPS) catalyze the production of geranyl diphosphate (GPP) and geranylgeranyl diphosphate (GGPP), respectively. GPP serves as a direct precursor of monoterpenoids, and GGPP acts as a direct precursor of diterpenoids, carotenoids, and gibberellin [65]. 1-deoxyd-D-xylose-5 phosphate synthase (DXS) acts as the rate-limiting enzyme in this process [66,67]. All of these precursors eventually generate various terpenoids through the catalysis of different terpene synthases [68]. In addition, studies have indicated that certain terpene synthases utilize neryl diphosphate (NPP) as a substrate precursor to generate terpenoids rather than GPP, GGPP, and FPP [69,70,71]. In general, the synthesis of terpenoids is divided into three key production stages, namely the generation of the C5 precursors IPP and DMAPP, the generation of the direct substrates GPP and FPP, and the generation of terpenoids. The first two stages have been basically studied as a common metabolic pathway for the synthesis of all terpenes, whereas the third stage is critical for terpene metabolism and determines the diversity of structures and species (Figure 2).

### 3.2. Research Advances in Terpene Synthase Genes

During the synthesis of volatile terpene, terpene synthases are located in the branch nodes of terpene anabolism and directly catalyze FPP or GPP to form various monoterpenes, sesquiterpenes, etc. Meanwhile, terpene synthases exhibit diverse catalytic activities, enabling the formation of multiple products from a single substrate. Their catalytic functions and efficiencies determine the diversity of terpenoid compounds, which subsequently influences the composition and content of plant volatiles. Consequently, terpene synthases are key enzymes in the formation of terpenes and are important determinants of plant fragrance characteristics.

Most terpene synthases have typical DDXXD conserved domains for binding divalent cations, such as Mg^2+^ and Mn^2+^ [72]. However, some terpene synthases involved in monoterpene synthesis may also feature an RRX8W conserved domain and a plasmid localization signal peptide at the N-terminus [73,74]. Terpene synthases are characterized by diverse types, structures, and functions. According to the similarity of their genome sequences, they can be divided into gymnosperm terpene synthases, angiosperm terpene synthases, etc. [75]. Alternatively, they can be classified by the type of catalytic products they generate, including monoterpene synthase, sesquiterpene synthase, diterpene synthase, and triterpene synthase [76]. More commonly, terpene synthases are grouped according to the similarity of their amino acid sequences into seven subfamilies, designated TPS a–g [77]. Members of the same subfamily are usually similar in terms of species classification or catalytic function. For example, the TPS-a subfamily primarily consists of angiosperm sesquiterpene synthases, while the TPS-d subfamily is almost exclusively composed of gymnosperm terpene synthases and TPS-g family members are usually bifunctional terpene synthases [78,79,80]. Recent studies have shown that the phylogenetic classification of terpene synthases is more closely related to species affinity, relatively distant to their catalytic products. Specifically, terpene synthases with similar functions often exhibit low sequence similarity across species, while those with different functions may show high sequence similarity within the same species [68]. For example, *Perilla* myrcene synthase and *perilla* limonene synthase show up to 90% amino acid sequence similarity despite their distinct catalytic products [81,82]. Conversely, the sequence similarity between *Arabidopsis* and *snapdragon* linalool synthase is as low as 42%, yet the sequence similarity between linalool synthase and nerolidol synthase in *snapdragon* reaches 95% [83]. This highlights the need for accurate operations, such as enzyme activity detection in vitro, to verify the specific catalytic function of certain terpenoid synthase proteins, rather than using single-sequence homology comparisons. Beyond catalytic properties, factors such as the subcellular localization of TPS expression and competitive affinity to substrates can also affect the type and proportion of products. For example, two nearly identical terpene synthases, AmNES/LIS-1 and AmNES/LIS-2, are compartmentally segregated due to their subcellular localization differences in snapdragon and catalyze nerolidol and linalool formation, respectively. Similarly, in *A. chinensis* flowers, the uncharacterized TPS protein responsible for acnesol biosynthesis appears to compete for the available pool of FDP utilized by AcNES1, indirectly and strongly influencing the amount of nerolidol released [78].

Since Facchini and Chappell first cloned two sesquiterpene synthase genes in tobacco in 1992 [84], numerous scientists have focused their efforts on studying terpene synthase genes. Up to now, the cloning and functional analysis of terpene synthase genes are still hot topics in plant molecular biology research, and numerous terpene synthase genes have been identified in many terrestrial plants, including the snapdragon [78], rose [84], mouse-ear cress [85], and tomato [86]. By comparison, it was found that more cloned *TPS* genes have been derived from core dicotyledonous plants, mostly from the vegetative tissues and fruits of cultivated species, while relatively few TPS genes were expressed specifically in flowers. The *TPS* gene resources from wild parent species and related species within the same genus need to be further explored. Recently, Yang Song et al. revealed the molecular mechanism of the synthesis of the main volatile terpene synthase in *Aquilegia* by analyzing the functions of key terpene synthase genes, and further explored the significant changes in catalytic activity or product specificity caused by non-synonymous mutations or the amino acid polymorphisms of terpene synthase genes [87]. Gao et al. cloned eight terpene synthase genes associated with volatile terpene emission in cultivated *Freesia* x *hybrida* Red River^®^ and Ambiance [88]. Bao et al. cloned 15 terpene synthase genes in eight wild *Freesia* species and further explored the changes in catalytic capacity caused by partial allelic variation, thereby jointly revealing the molecular basis of the synthesis of volatile terpenoid diversity between members of the *Freesia genus* [89].

Terpene synthase genes are pivotal in terpene synthesis, and their expression level and catalytic activity directly determine the diversity of terpenes in plant volatiles. Studies have shown that many terpene synthases have diverse functions, capable of producing single or multiple terpenes from the same substrate or utilizing different substrates to simultaneously catalyze the production of monoterpenes and sesquiterpenes. Additionally, the subsequent modification of reaction products can also affect the richness of volatiles. Moreover, minor structural changes in some terpene synthases may lead to changes in their catalytic functions and produce new catalytic functions [90]. This phenomenon may contribute significantly to terpenoid diversity and the complexity of regulating terpenoid synthase gene expression.

## 4. Biosynthesis Regulation of Plant Terpenoids

The synthesis and release of plant volatile terpenoids represent a stress strategy, enabling plants to cope with external environmental changes that are regulated by multiple factors, including the plant’s developmental stage or tissue specificity, abiotic factors (light, temperature, and humidity), and biotic factors (insect gnaws and pathogenic microorganisms). Furthermore, extensive research indicates that the synthesis of terpenoids is mostly regulated by transcription factors.

### 4.1. Environmental Factors Affecting the Biosynthesis of Plant Terpenoids

The synthesis and volatilization of plant terpenoid volatile compounds exhibit spatiotemporal specificity and are typically released in large quantities through specific tissues, such as stamens and glandular hairs, during specific developmental stages, including flower opening and fruit ripening. The flowers of plants are particularly rich in volatile terpenes. A significant correlation between terpene emission and the opening period of Freesia flowers, the release of terpenoids, and the expression of related structural genes increased gradually with the blooming of flowers [88]. The primary components of the flower fragrance of snapdragon, myrcene and ocimene, rapidly increase from its upper and lower lip flap on the second day after flowering and reach their peak on the sixth day [78]. Glandular hairs are important organs for synthesizing, storing, and releasing terpenoids in many plants, such as tomatoes and tobacco. The monoterpene content in mint leaves varies across different development stages, with monoterpenes accumulating rapidly in the first 21 days of early leaf development, before remaining stable [91,92]. In addition, the growth and developmental states of plant leaves influence the volatilization rate of terpenes. The young leaves of eucalyptus, peppermint, and Artemisia release more pinene and terpenes than older leaves [93,94].

The synthesis and release of terpenoids are influenced by both biotic and abiotic factors. Intense light stimulates the synthesis of menthofuran in large quantities, whereas elevated temperature and humidity enhance the synthesis and release of monoterpenoids. *Rosmarinus officinalis* releases significantly more monoterpenes during the high-temperature season compared to other seasons [95]. The volatilization rate of *Pinus elliotii* was proportional to the leaf’s surface temperature [96]. The effect of high humidity on the release of monoterpenoids is likely mediated by its impact on stomatal opening and closing. When the air humidity drops below 40%, the release rate of *Pinus ponderosa* decreases sharply [97]. The effect of light on the release of terpenoids varies with different plant species. Some monoterpenoids volatilized from young conifer leaves are related to light intensity. *Hieronyma* spp. and some species of Quercus emit monoterpenoids in a light-dependent manner [98]. Water scarcity significantly reduces the monoterpene and sesquiterpene substances in plants such as rosemary [99]. Consequently, seasonal changes related to light intensity, temperature, and humidity can significantly affect the release of monoterpenes in plants.

The increased release of volatile substances from animal and insect gnawing is well-documented and widely recognized. For example, forest caterpillar gnawing induces the expression of terpenoid synthase genes and promotes the release of terpenoids in poplar leaves [40].

These findings have been elaborated upon in previous studies and will not be detailed further here. In addition, during the process of synergistic evolution with pollinators, some plants exhibit circadian rhythms in flower opening and terpene release to match pollinator schedules. In general, flowers pollinated by daytime active insects, such as bees and butterflies, release significant quantities of volatiles during daylight hours, while those with moths, bats, and other nocturnal insects as pollinators emit larger amounts of terpene volatiles during the night [28].

### 4.2. Current Status of the Transcriptional Regulation of Plant Terpenoid Biosynthesis

Early studies predominantly concentrated on the cloning and functional characterization of enzymes in terpenoid metabolic pathways. In recent years, with the rapid development of molecular biology technology, scientists have focused more attention on the transcriptional regulation of terpenoids. Transcription factors, as upstream regulatory elements of plant secondary metabolic pathways, simultaneously regulate several key enzymes in downstream metabolic pathways, thus efficiently promoting the generation of target substances [100]. Therefore, studying and refining the regulatory network of terpenoid metabolism holds significant potential for engineering applications, offering more effective strategies for the development and utilization of high-value plant-derived terpenoids.

Transcription factors, also known as trans-acting factors, are DNA-binding proteins capable of binding to specific regions of the eukaryotic gene promoter region to regulate gene expression [101]. Statistical analyses reveal that 58 distinct types of transcription factors are involved in the regulation of plant secondary metabolism [102]. As early as 2002, Croteau et al. proposed that transcription factors might be involved in the regulation of terpenoid synthesis, but relevant studies have not received enough attention [103]. In recent years, the rapid development of molecular biology techniques, particularly omics techniques, has intensified the focus on the transcriptional regulation of terpenoid synthesis. It can be inferred that the transcriptional regulation of the synthesis of volatile terpenoids is significantly different from that of other plant secondary metabolites. For example, the synthesis of anthocyanins and proanthocyanins depends on a ternary MBW regulatory complex composed of MYB/bHLH/TTG1 [104], while the synthesis of flavanols is mainly regulated by the MYB transcription factor alone [105]. In contrast, a wide variety of transcription factors regulate the expression of *TPS* genes (Table 2). Currently, ten types of transcription factors, including AP2, NAC, bZIP, WRKY, SBP, ARF, SRS, HSF, MYB, and bHLH, have been identified in a variety of plants, such as periwinkle, *Arabidopsis thaliana*, mint, corn, cotton, tomato, *Artemisia annua*, tobacco, kiwi, and citrus, as being involved in the regulation of terpenoid metabolism (Table 2) [64,106,107,108,109,110,111,112,113,114,115,116,117,118,119,120,121,122,123,124,125,126,127,128,129,130,131,132,133,134,135,136,137,138,139,140].

Transcriptional regulation of terpenoid synthesis is a highly complex process, with multiple transcription factors having been reported to be involved in terpenoid anabolism, even in the same species. Three types of transcription factors, including NAC, WRKY, and HSF, have been identified as regulating terpenoid anabolism in *Gossypium hirsutum* [125,139,140]. In the tomato, SlEOT1, SlMYC1, and SlWRKY73 were reported to activate the transcription of the *TPS5* promoter of the linalool synthase gene [125,138]. In addition to the direct regulatory role in controlling the transcription levels of genes related to terpenoid synthesis by individual regulators, relatively few hierarchical regulations, as well as synergistic or antagonistic effects, have been elucidated among different transcription factors. *AtMYB21* was experimentally verified as being underexpressed in an *arf6 arf8* mutant, consistent with the dramatically fewer sesquiterpenes produced by TPS11 and TPS21 [118]. The consistently highly expressed AtMYC2 interacts with AtMYB21 to enhance the expression of sesquiterpene synthase genes *TPS11* and *TPS21*, although it negatively affects the activation of the monoterpene synthase gene *TPS14* by AtMYB21, which takes concerted action, with GC-MS detection results of over 90% for the sesquiterpene caryophyllene and extremely trace amounts of the monoterpene linalool in *Arabidopsis thaliana* [134]. A similar synergistic effect between FhMYB21 and FhMYC2 was also demonstrated in *Freesia*, although FhMYC2 exhibited a more discrepant inhibitory effect than AtMYC2 on sesquiterpenoids overall due to its significantly reduced transcriptional activation activity [134]. The latest study, utilizing single-cell sequencing technology, found that GoHSFA4a and GoNAC42 can directly regulate the expression of genes involved in the biosynthesis of terpenoids in secreted adenocytes of *Gossypium hirsutum* in response to developmental and environmental stimuli [140].

## 5. Targeted Engineering of Terpenoid Mass Production

With the rising demand for an improved quality of life, individuals are becoming increasingly resistant to products from chemical mass production labeled as unnatural or artificial. Relevant regulations are also restricting the unscrupulous abuse of these chemicals, and the demand for natural terpenoid products is increasing [141]. Collectively, these factors have become important driving forces for the popularity of natural terpenoid products. A prime example is the concept of essential oils, primarily composed of plant terpenoids, which has been warmly embraced by people and dubbed as “liquid gold” due to its high economic value. The extensive application of terpenoids in food flavors, perfumes, pharmaceutical preparations, and pest control has rendered them a highly desirable commercial product. Therefore, a lot of effort and investment has been made to construct plant and microbial platforms for their advanced industrial overproduction.

**Figure 3:**
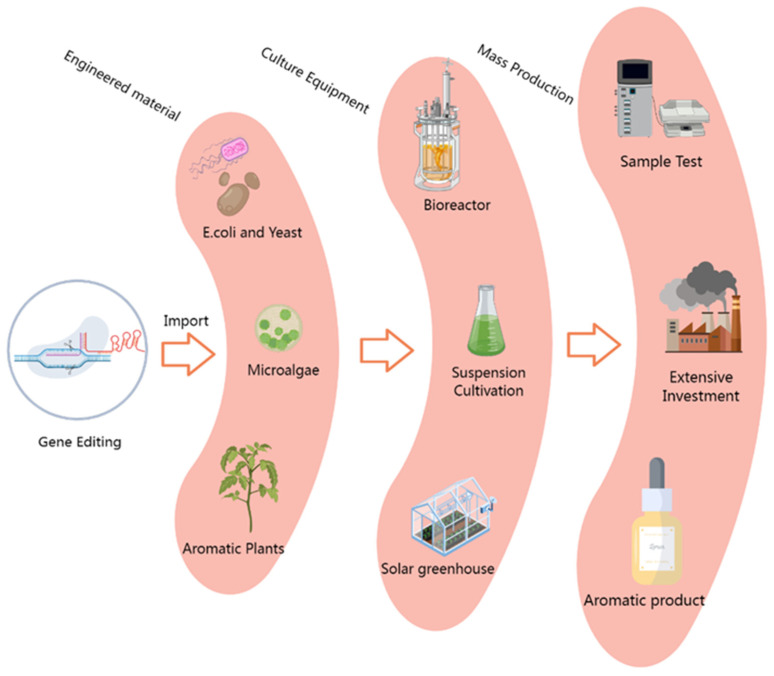
Microorganism- and plant-based target engineering for the mass production of volatile terpenoids. Engineering vectors containing key genes for terpenoid synthesis were introduced into microorganism, plant, or cyanobacteria bioreactor platforms through optimized transformation methods, and after adaptive laboratory culture, high-yield target component production microsystems were obtained. After component testing, extraction, and culture program optimization, it is expected to reduce production costs by great strides and achieve the large-scale industrial production of terpenoids.

Microbes, particularly *E. coli* and yeast, exhibit significant advantages in metabolic engineering (Figure 3), such as implementable gene editing and transformation procedures, rapid growth and reproduction, convenient harvesting of secretory metabolites, and inexpensive nutrient sources. Another major advantage that cannot be ignored is the minimal presence of competing branches and the low probability of feedback inhibition in microorganisms during plant metabolite production [142,143,144]. Hence, microbes are regarded as an ideal platform for engineering the overproduction of plant terpenoids, and considerable efforts have been put into realizing and optimizing this system. In addition, secondary metabolites produced by microbial engineering are considered healthy and highly acceptable to the general public, such as microbial-produced vanillin, which is already commercially available [145,146]. However, using microbial engineering to produce terpenoids still has many limitations that need to be addressed. Commonly used engineering microbes, such as *Escherichia coli* and yeast, lack a complete terpenoid anabolic pathway, necessitating the empirical heterologous expression of many key enzyme genes within these strains. This may require silencing or modifying specific genes to direct metabolic flow towards target products or removing toxic metabolites, which is hampered by progress related to target metabolite synthesis pathways. Another challenge arises from the expression of exogenous genes, which are often expressed in excess without forming the optimal configurations for the efficient synthesis of target metabolites. Excessive accumulation of metabolic intermediates, such as IPP and DMAPP, or side reactions from overexpressed components have been shown to obstruct other metabolic pathways in vivo, even causing a total breakdown if the concentration exceeds the threshold levels [147]. Therefore, future research on terpenoid overproduction still needs to focus on anabolic pathways, regulatory mechanisms, and the functional analysis of key enzymes to provide gene pools for microbial synthesis engineering and optimize configurations for efficient production through multi-disciplinary integration, such as synthetic biology and biostability dynamics.

Initially, plants were considered unsuitable for the factory production of terpenoids due to several disadvantages, such as long growth periods, expensive extraction costs with poor yields, and complicated gene transformation procedures. However, as understanding of metabolic flows within or between target pathways, molecular regulatory mechanisms, big data gene function analysis, etc., has improved, especially the gradual maturity and application of fine genome-editing tools and transgenic technologies, plants have been shown to be an attractive system for the overproduction of target metabolites [148]. Economically, plant platforms are more cost-effective than microorganisms due to their autotrophic growth processes and lack of need for culture media and specialized equipment [149]. The metabolic pathway or modification of specialized metabolites may not have been fully deciphered, and plants are able to harvest target products while ignoring these uncharacterized reactions [150,151]. In the context of terpenoid biosynthesis, plants possess complete basic components to efficiently synthesize, store, and release terpenoids by enhancing the expression of multiple genes in the synthesis pathway and controlling metabolic flow through the introduction of specific regulatory factors or external stimuli, which is currently the most routine approach in plant metabolic engineering [152]. Despite these advantages, from an industrial perspective, plants lag behind microorganisms in the production of terpenoids, as evidenced by the fact that no commercially available terpenoids derived from engineered plants have yet been found on the market, even though there are numerous reports of altering flavor and nutritional qualities through gene editing. The primary contributing factor to this situation is the limitation of advanced molecular tools, such as target gene cell localization technology and instantaneous virus transformation technologies, which are mainly used in theory and scientific research rather than molecular agriculture. Additionally, current regulations and public acceptance have exacerbated this gap, as most genetically modified plants and their derived products are subject to complex regulations and are perceived as risky by the public. Moreover, plant terpenoid engineering is more complex than that of microorganisms due to the intricate symphony of its internal metabolic branches, making it extremely complicated to increase metabolic flux to this energy pool, particularly when intersection nodes between distinct branches compete for the same substrate or affect other important agricultural traits (Figure 3). The scale of this phenomenon is easy to expand, potentially producing profound effects on the external environment and changing the homeostasis of plant–pollinator interactions. Therefore, engineering improvements require elaborate designs to ideally consider integrating the understanding of the various aspects of plant terpenoid interactions with the environment. Notably, microalgae and plant cell bioreactors combine the advantages of plants and microbes and are expected to become excellent metabolic engineering systems, warranting further research efforts in the coming years [153,154].

Both the “harvest without planting” approach in microbial engineering and the “directed cultivation” approach in plant engineering, which targets active ingredients, possess significant potential for producing target terpenoids. Further improvement and development of these approaches will require in-depth analysis of metabolite synthesis pathways and regulatory mechanisms, along with continuous integration of biosynthesis, systems biology, and molecular genetics technologies, thereby contributing to the maintenance and provision of human well-being.

## 6. Conclusions

Volatile terpenoids are important secondary metabolites in plants. They not only play a significant role in the plant defense process but also serve a crucial function in responding to changes in the external environment and participating in signal communication with surrounding organisms. In addition, plant volatile terpenoids exhibit great application potential in aspects such as insect resistance, disease resistance, antibacterial activity, healthcare, and food preservation.

The synthesis of terpenoids in plants has been thoroughly analyzed. Among them, terpenoid synthase genes play an important role in determining the diversity of the final products. The synthesis of terpenoids is regulated by many factors, including environmental factors and key transcription factors. Currently, as many as 10 transcription factors involved in the regulation of terpenoid synthesis have been reported, and the transcription factors playing key regulatory roles reported in different species vary significantly. This may also be an important factor contributing to the huge differences in the volatile terpenoid fingerprints of different plants.

The widespread application of terpenoids has promoted the rapid emergence of targeted terpenoid production engineering. Microorganisms and plants show great application potential in the rapid production of terpenoids. This article comprehensively reviews the research progress on the functions, synthetic pathways, key genes, regulatory factors, and targeted engineering of volatile terpenoids, which will provide important theoretical support for the engineering production of terpenoids and the molecular breeding of target terpenoid plants.

## Figures and Tables

**Table 2:** Transcription factors that regulate the production of volatile terpenoids in plants.

Gene Family	Transcription Factor	Species	References
**MYB**	PtMYB14	*Pinus taeda*	[106]
	AtMYB21	*Arabidopsis thaliana*	[107]
	FhMYB108, FhMYB21L1/2	*Freesia hybrida*	[26,108]
	AaMYB1	*Artemisia annua*	[109]
	SlMYB75	*Solanum lycopersicum*	[110]
	PbMYB21/61	*Betula platyphylla*	[111]
	MSMYB	*Mentha spicata*	[112]
	JsMYB305/108	*Jasminum sambac*	[64]
	LiMYB305	*Lilium brownii*	[113]
	LaMYB1	*Lavender*	[114]
	HcMYB1/2/7/8/75/145/248	*Hedychium coronarium*	[115,116]
	AmMYB24	*Antirrhinum majus*	[117]
**bHLH**	AtMYC2	*Arabidopsis thaliana*	[118]
	SlMYC1	*Solanum lycopersicum*	[119]
	PbbHLH4/6	*Phalaenopsis orchids*	[120]
	bHLH9	*Betula platyphylla*	[121]
	LoMYC2	*Lilium brownii*	[122]
	FhMYC2	*Freesia hybrida*	[108]
	CpMYC2	*Chimonanthus praecox*	[123]
	LaMYC7	*Lavender*	[124]
**WRKY**	GaWRKY1	*Gossypium hirsutum*	[125]
	AaWRKY1	*Artemisia annua*	[126]
	SlWRKY73	*Solanum lycopersicum*	[127]
	OfWRKY7	*Osmanthus fragrans*	[128]
**AP2/ERF**	EREB58	*Zea mays*	[129]
	ERF2, ERF61	*Osmanthus fragrans*	[130,131]
	AaERF1/2	*Artemisia annua*	[132]
	CitERF71, CitAP2.10	*Citrus sinensis*	[133,134]
**ARF**	HcARF5	*Hedychium coronarium*	[135]
**bZIP**	bZIP1, HY5	*Artemisia annua*	[136,137]
**SRS**	SlEOT1	*Solanum lycopersicum*	[138]
**SBP**	AtSPL9	*Arabidopsis thaliana*	[139]
	SPL3/9/10	*Pogostemon cablin*	[139]
**NAC**	GoNAC42	*Gossypium hirsutum*	[140]
**HSF**	GoHSFA4a	*Gossypium hirsutum*	[140]
